# Thanks to Reviewers 2023

**DOI:** 10.5334/pb.1289

**Published:** 2024-01-25

**Authors:** Editorial Team

**Affiliations:** 1 Psychologica Belgica, Belgium

## Abstract

All manuscripts published in Psychologica Belgica have been assessed conscientiously and unselfishly by expert reviewers. The quality of our journal totally depends on their valuable and constructive criticisms to the authors. Both the editors and the authors highly appreciate the input and dedication of all our reviewers. Many thanks.

Lorenzo Avanzi

Abdel-Halim Boudoukha

Damien Brevers

Athena Cairo

Manon Couvignou

Catherine Culot

Nele De Cuyper

Jonas De keersmaecker

Kobe Desender

Kim Dierckx

Federica Farolfi

Wim Fias

Aurore Gaboriaud

Saba Ghayas

Pol Ghesquiere

Medhi Gilson

Danielle Godon-Decoteau

Michel Hansenne

Van Grieken Katrien

Olivia Kirtley

Mikhail Kissine

Evy Kuijpers

Ouwens Machteld

Vandecandelaere Machteld

Pierre Maurage

Xavier Noël

Santos Orejudo

He Peng

Martine Poncelet

Elodie Pools

Etienne Quertemont

Cort Rudolph

Thorsten Rudroff

Ehri Ryu

Michiko Sakaki

Marie-Anne Schelstraete

Sarah E Schimschal

Julia Schnepf

Dirk Smits

Céline Stassart

Andreas Stengel

Jaimee Stuard

Arnaud Szmalec

Ieva Urbanaviciute

Laurens Van Calster

Stefaan Van Damme

Chantal van den Berg

marius van dijke

Florian van Leeuwen

Nette Vandenhouwe

Tim Vanhoomissen

Leentje Vervoort

Ivana Vranjes

Joachim Waterschoot

Aliza Werner-Seidler

Keith Widaman

Rick Wilson

